# Dental fluorosis among people and livestock living on Gihaya Island in Lake Kivu, Rwanda

**DOI:** 10.1186/s42522-021-00054-7

**Published:** 2021-12-20

**Authors:** Theodore Habiyakare, Janna M. Schurer, Barika Poole, Susan Murcott, Basile Migabo, Birori Mardochee, J. Hellen Amuguni, John P. Morgan

**Affiliations:** 1 Center for One Health, University of Global Health Equity, Butaro, Rwanda; 2 Cummings School of Veterinary Medicine at Tufts University, North Grafton, USA; 3 Massachusetts Institute of Technology, Boston, USA; 4 BRP Consulting Inc., Jacksonville, USA; 5 College of Medicine and Health Sciences, University of Rwanda, Kigali, Rwanda; 6 College of Science and Technology, University of Rwanda, Kigali, Rwanda; 7 School of Dental Medicine, Tufts University, Boston, USA

**Keywords:** Fluoride, Dental fluorosis, Rwanda, One Health, WASH

## Abstract

**Background:**

Dental fluorosis is caused by prolonged exposure to excessive fluoride during the period of permanent tooth formation and is characterized by tooth discoloration, pitting, and loss of shape. Communities living near Lake Kivu in Western Rwanda exhibit a high prevalence of dental fluorosis; however, data on prevalence and risk factors are scarce.

**Methods:**

This cross sectional, quantitative study used a One Health approach to investigate dental fluorosis prevalence among people and livestock and to measure fluoride content in the environment. In 2018, oral health examinations were conducted to assess the prevalence of fluorosis in children (aged 9 to 15 years), cattle and goats residing on Gihaya Island (Rwanda, East Africa). All children and cattle/goats meeting basic eligibility criteria (e.g., island residence) were invited to participate. Presence and severity of dental fluorosis was categorized according to the Dean’s Fluorosis Index. Samples of local foods, water, soil and grass were collected from communal sources and individual households and analyzed for fluoride content using standard laboratory techniques. Descriptive and binomial analyses (Fisher Exact Test) were used to assess this dataset.

**Results:**

Overall, 186 children and 85 livestock owners (providing data of 125 livestock -23 cattle and 102 goats) participated. Dental fluorosis was recorded in 90.7% of children and 76% of livestock. Moderate to severe fluorosis was observed in 77% children while goats and cattle most often exhibited mild or absent/questionable severity, respectively. Water from Lake Kivu (used primarily for human cooking water and livestock drinking water) contained fluoride levels that were consistently higher than the maximum threshold (1.5 mg/L) recommended by the World Health Organization. Other sources (borehole and rainwater) were within safe limits. All food, soil and grass samples contained fluoride. The highest levels were observed in porridge (0.5 mg/g) and small fishes (1.05 mg/g).

**Conclusions:**

Altogether, dental fluorosis was highly prevalent among children and goats on Gihaya Island with various food and water sources contributing a cumulative exposure to fluoride. An immediate and coordinated response across human, animal and water professionals is needed to reduce fluoride exposure within safe limits for island residents.

**Supplementary Information:**

The online version contains supplementary material available at 10.1186/s42522-021-00054-7.

## Introduction

Fluoride is a naturally occurring element that contributes to the formation of mineralized structures such as teeth and bone [1]. Excessive ingestion of fluoride can result in dental and skeletal fluorosis among people and a range of domestic animals [1–4]. Dental fluorosis is an enamel mineralization disorder caused by long period of high intake of fluoride during early stages of tooth formation [1]. Dental fluorosis can result in tooth discoloration, pitting of tooth enamel and weakening and collapse of tooth structure [5]. High fluoride exposure can also cause skeletal fluorosis, characterized by joint pain and/or stiffness, bone deformities and fractures [6]. Since the 1930’s, dental fluorosis has been used a biomarker in humans to indicate systemic fluoride exposure [7].

Water is the most important source of dietary fluoride in people, accounting for 75–90% of daily intake [8]. At moderate water fluoride levels (0.5 to 1.0 mg/L), fluoride protects teeth against development of dental caries and is not known to cause adverse systemic affects [9]. Prolonged exposure to water fluoride levels above this maximum permissible level can result in dental fluorosis (1.5 mg/L), skeletal fluorosis (3.0–6.0 mg/L) and crippling fluorosis (10 mg/L) in people [10, 11]. Groundwater fluoride levels above 1.5 mg/L have been associated with severe dental fluorosis in cattle (*Bos taurus*), buffalo (*Bubalus bubalus*), and goats (*Capra aegagrus hircus*) [2, 3]. Higher levels (2.8 mg/l and above) are associated with skeletal fluorosis, characterized by stiffness, bony exostoses and hind limb lameness [12]. Some foods, such as tea leaves, curly kale, fish, and food tenderizers (magadi) can also contain high levels of fluoride and contribute to excessive fluoride exposure in people [13–15]. Risk factors for animals include excessive fluoride levels in soil where animals’ fodder is grown and fluoride from industries [16].

Excessive fluoride exposure during early growth and development influences the extent and impact of dental fluorosis. Children between one and four years of age are at the highest risk for dental fluorosis in their permanent anterior teeth [17]. After eight years of age, the permanent anterior teeth are generally fully developed and less subject to esthetic change [18]. The exposure of juvenile animals to excessive fluoride during teeth formation period is implicated in dental fluorosis of anterior teeth [19]. In human populations, loss of tooth esthetics due to dental fluorosis is associated with poor self-esteem, shame, and stigma [20].

In 2017, the National Oral Health Survey of Rwanda conducted country-wide oral health examinations and determined that 93 of the 126 identified dental fluorosis cases lived on Gihaya Island in the Western Province of Rwanda [21]. Data pertaining to scope and risk factors of dental fluorosis in Gihaya Island and Rwanda are scant. Therefore, this study aimed to: (a) assess the prevalence of fluorosis in children with permanent anterior dentition (b) assess the prevalence of fluorosis in livestock (c) identify dietary sources of fluoride for humans and livestock and (d) measure fluoride levels of environmental samples on Gihaya Island. Both people and animals require safe drinking water for optimal health; moreover, rural farming communities in Rwanda rely on health livestock (in this case, goats and cattle) for animal-source protein and household income. The strong relationship between water safety and dental fluorosis among people and animals indicate that this is a One Health issue requiring a multi-disciplinary and inter-sectional approach.

## Materials and methods

### Study setting

Gihaya Island is a small island situated in Lake Kivu between the Democratic Republic of Congo and Rusizi District in Western Rwanda. The ~ 1,230 human residents are almost exclusively engaged in subsistence agricultural activities, growing staple crops, harvesting small fish, and raising livestock (goats, chickens, cattle, pigs) to feed their families and earn income. Three boreholes exist but only one solar-powered water pump was operational at the time of this study. Basic health services can be accessed through Community Health Workers and a local health post. There are no oral health services on the island.

### Participant recruitment

Data for this study were obtained through (1) interviewer-administered surveys of children and livestock owners, (2) oral examinations of children and livestock, and (3) food, water, and environmental sample collection and analysis. Inclusion criteria for children were ages 9–15 years, residence on Gihaya Island since birth, upper and lower anterior permanent central and lateral incisors fully erupted, and parental permission to participate in the study. For inclusion of livestock data, owners were required to be 18 years or above. Goats and cattle were required to be over six months of age, have resided on the Gihaya Island since birth and able be handled safely (i.e., animal remained calm without chemical sedation). The inclusion criteria were developed to allow for the maximum number of permanent teeth in children and animals who were lifelong residents of the Island. All eligible children and livestock living on this island were invited to participate. Surveys, oral health assessments and initial food sampling were conducted over a four-day period in December of 2018. Community residents were informed of the study by local Community Health Workers prior to and on the day of data collection. All eligible children and livestock owners (with their livestock) were invited to participate. Surveys and oral examination of children were conducted at the local primary school while livestock owners were met at their homes. Based on high levels of human dental fluorosis and water fluoride content observed in 2018 (as reported in this study), a second research team returned to the island in August of 2019 to collect additional water and food samples for fluoride and microbial analyses, and to measure assess dietary habits (A. Heiman, unpublished data). To our knowledge, there were no relevant social or environmental changes impacting natural water sources during the time interval.

### Study tools

The questionnaire for children addressed demographics, water sources, diet, and satisfaction with tooth appearance (Additional file 1). The questionnaire for livestock owners addre ssed owner/livestock demographics, livestock water sources, and livestock food (Additional file 2). All questions were yes/no or multiple choice and were modified from previously published resources to be contextually relevant for Gihaya Island [22, 23]. Questionnaires were developed in English, translated into Kinyarwanda, and pretested for face and content validity.

Oral examinations of children assessed the presence of decayed missing and filled teeth and the presence and severity of fluorosis on maxillary and mandibular permanent anterior teeth (fully erupted central incisors, lateral incisors and cuspids) [24]. The presence and severity of fluorosis were also recorded for lower permanent anterior teeth of goats and cattle. The Dean’s Fluorosis Index [25] was used to visually classify fluorosis severity in children and a modified version (absent, mild, moderate, severe) was used to evaluate livestock. Oral examinations in children and livestock were conducted by a licensed dental therapist and licensed veterinarian, respectively, using headlamps for illumination when necessary. Children were comfortably seated in a chair and the teeth dried with a 2 × 2 gauze. When fluorosis presence or severity was doubtful, the lower of the two categories under consideration was assigned. Photographs were taken and examined at the end of the study to ensure consistent fluorosis categorization. Questionnaire and examination data were directly entered into Kobo Toolbox using cellular smartphones [26].

### Water, food, grass and soil sampling

Initial water samples (2018) were obtained near the lake shore where residents collected cooking and drinking water, from the one functional borehole and from the local health post rainwater collection tank. Three samples were collected at each location using standard collection techniques for wading. Samples from each location were tested in triplicate with the fluoride testing probe at the University of Rwanda laboratory in Kigali. Samples of locally sourced and commonly eaten uncooked foods (cassava root, cassava leaf, bean leaf, fresh beans, dried beans, and small fishes) were collected from island residents. Soil and grass samples were collected from two livestock grazing points on the island. One sample of each food, grass and soil type were collected. In 2019, three additional water samples were collected from each of the following water sources: six locations around the island where people gather water for drinking and cooking from Lake Kivu, a water storage tank from a solar-powered borehole pump, and a rainwater catchment tank at the local health post. Various prepared foods (beans, green bananas, ugali, green marog, small fishes, and porridge) were collected from two households, which had cooked foods with water from Lake Kivu according to their norm. All water, food, soil, and grass samples were collected under the supervision of a certified lab technician and stored in sterile 100 mL plastic bottles or plastic zip lock bags. Samples were transported in cooled containers and stored in refrigerators until analyzed.

### Fluoride concentration measurement

Food and soil samples were dried in an oven at 105 °C for 12 – 24 h. Dried sample were crushed into a fine powder with a mortar and pestle. For each sample, 1 g of powder was placed in a crucible and covered with 5 mL of NaOH 8 M solution. Crucibles were heated on a hot plate for a minimum of 5 min and then placed in an 200 °C oven for 16 h. After 16 h, crucibles were cooled to room temperature and then rehydrated in 15 mL distilled water. Solutions were gently shaken until homogenous, neutralized with 37% hydrochloric acid to reach pH 7.2 – 7.5, and then diluted in 25 mL distilled water before measuring the fluoride concentration.

Fluoride concentrations were measured using an pH/ISE ion selective electrode, with a fluoride probe (model ISEF12101) from HACH (Colorado, USA), according to the user manual instructions [27]. The fluoride probe was calibrated using 3 standard solutions (0.5; 1 and 2 mg/L) prior to fluoride analysis. Each raw water sample was measured three times. To test the effect of boiling water on fluoride concentration, raw water samples were boiled briefly and then re-tested. Three preparations of each food, soil and grass sample were measured. Mean fluoride levels were recorded in mg/L for water samples and mg/g in food and soil samples.

### Statistical analysis

Questionnaire data were uploaded from the KOBO platform and analyzed using Statistical Package for the Social Sciences (SPSS), version 25 (IBM Corp., Armonk, N.Y., USA). Descriptive statistics, including counts and percentages for categorical variables and means and standard deviations (SDs) for continuous variables, were calculated. The Fisher Exact Test was used to identify associations between categorical clinical outcomes and demographic variables of interest. A *p*-value of < 0.05 was considered statistically significant.

## Results

### Demographic information

Overall, 186 children (100% of eligible residents) participated in this study; 47.3% of the children were male while 52.5% were female (Table 1). The mean age was 11.9 years. Among 182 households on Gihaya Island, 85 owned goats and/or cattle meeting the inclusion criteria, and all agreed to participate. In total, 125 livestock were examined, including 102 goats and 23 cattle. Nearly all animals were female (97.6%) and three quarters (74.4%) were aged 1 year and above. Livestock owners were predominantly female (69.4%) and most (84.7%) considered themselves the primary caretaker of the animals examined.

**Table 1:** Prevalence of dental fluorosis among humans (*N* = 186), cattle (*N* = 23) and goats (*N* = 123) on Gihaya Island, Rwanda, 2018 (based on the Dean’s Fluorosis Index)

	**Absent/Questionable**	**Very mild**	**Mild**	**Moderate**	**Severe**	**Total**	*p*–value*
	**n (%)**						
*Humans*							
Gender^a^							
Male	9 (10.3)	3 (3.4)	8 (9.2)	48 (55.2)	19 (21.8)	78 (89.6)	0.9
Female	8 (8.3)	3 (3.1)	11 (11.5)	57 (59.4)	17 (17.7)	88 (96.6)	
Total	17 (9.3)	6 (3.3)	19 (10.4)	105 (57.4)	36 (19.7)	166 (90.7)	
*Livestock*							
Type							
Goats	11 (10.8)	NA	74 (72.5)	17 (16.7)	0 (0)	91 (89.2)	< 0.001
Cattle	19 (82.6)	NA	4 (17.4)	0 (0)	0 (0)	4 (17.4)	
Total	30 (24.0)	NA	78 (62.4)	17 (13.6)	0 (0)	95 (76.0)	
Sex							
Male	1 (33.3)	NA	1 (33.3)	1 (33.3)	0 (0)	2 (66.4)	0.3
Female	29 (23.8)	NA	77 (63.1)	16 (13.1)	0 (0)	93 (76.2)	
Total	30 (24.0)	NA	78 (62.4)	17 (13.6)	0 (0)	95 (76.0)	

*NA* = Not Applicable due to modification in Dean’s Fluorosis Index for livestock

^*^ Determined using Fisher’s Exact Test

^a^ Data regarding gender were available for 183 out 186 participants

### Oral evaluation

**Fig. 1:**
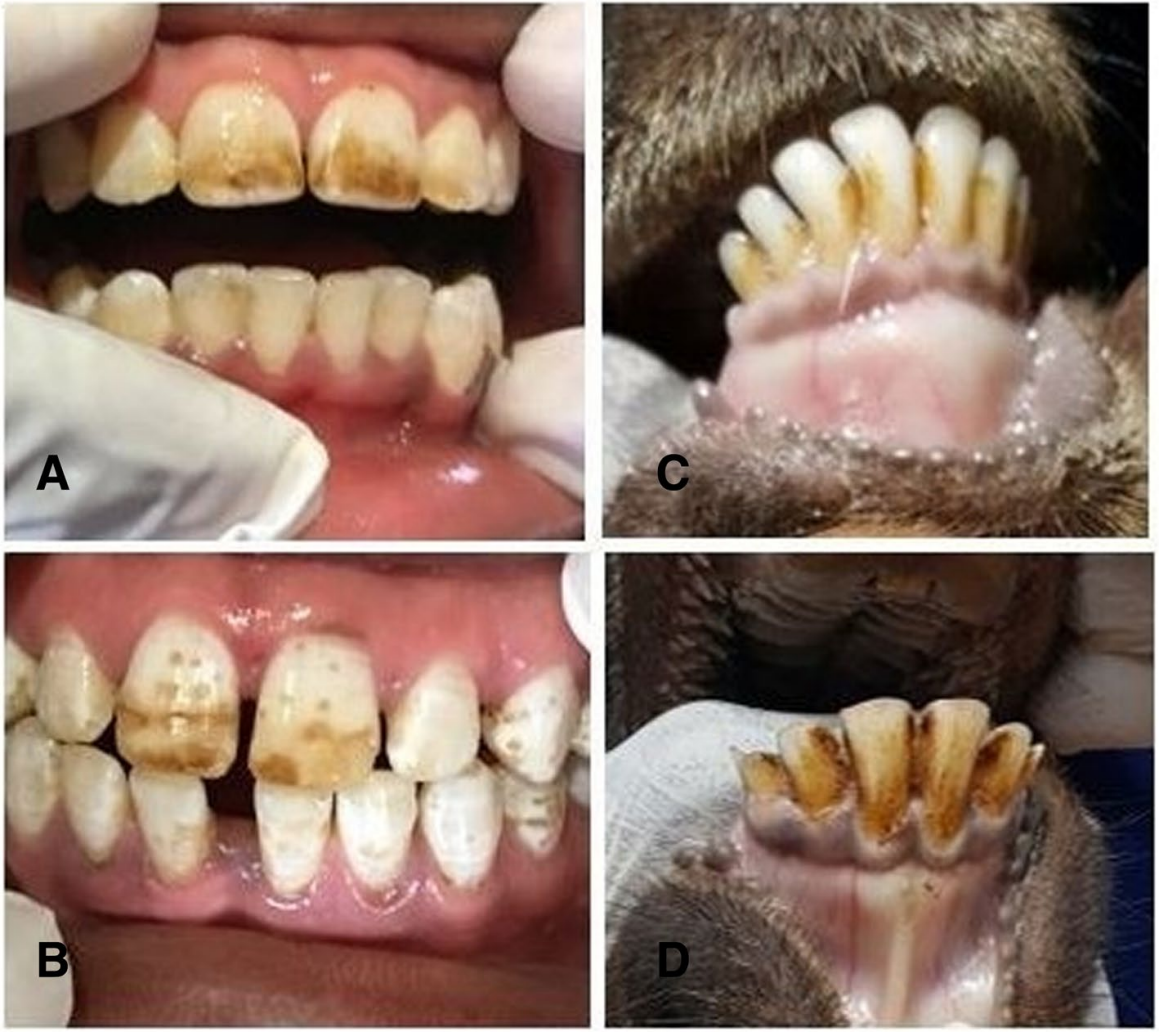
Dental fluorosis in humans and goats on Gihaya Island (2018). **A** Child with moderate dental fluorosis, **B** Child with severe dental fluorosis, **C** Goat with mild dental fluorosis, **D** Goat with moderate dental fluorosis

Nearly all (90.7%) children had signs of dental fluorosis, with most (77%) categorized as moderate to severe (Fig. 1). There was no statistical difference in prevalence between males and females (Table 1). When asked if they were satisfied with their tooth appearance (yes/no), three quarters (74%) of children answered in the negative. The prevalence of fluorosis among livestock was 76% and varied significantly (*p*- value < 0.001) between goats (89.2%) and cattle (17.3%). Only mild fluorosis was present in cattle. Mild and moderate fluorosis was present in goats.

### Water and food sources

Most (95.7%) children stated that their households used the solar powered borehole as the main source of drinking water. Three-quarters (74.2%) reported Lake Kivu as the main household source of cooking water (Table 2). Livestock owners most often obtained water for their animals from Lake Kivu (52.9%), followed by the borehole (24.7%) and multiple sources (22.2%). Among the surveyed livestock owners, 70 (82.4%) supplemented their animals’ diets with household food scraps.

**Table 2:** Reported water sources for people and livestock on Gihaya Island, Rwanda (2018)

		**Water source**	**Frequency, n (%)**
Humans (*N* = 186)	Drinking water	Lake Kivu	8 (4.3)
		Borehole	178 (95.7)
	Total		186 (100)
	Cooking water	Lake Kivu	138 (74.2)
		Borehole	48 (25.8)
	Total		186 (100)
Livestock^a^	Drinking water	Lake Kivu	45 (52.9)
		Borehole	21 (24.7)
		Multiple sources	19 (22.4)
	Total		85 (100)

^a^*N* = 85 livestock owners

### Fluoride concentrations in food, soil, and water

Fluoride was present in all drinking and cooking water sources, as well as food, soil, and grass samples. Across 2018 and 2019, the fluoride levels measured in Lake Kivu (range: 1.64–1.75 mg/L) were higher than borehole water (0.13 mg/L) or rainwater (1.88 × 10^–3^ – 0.02 mg/L; Table 3). Mean fluoride concentration across all water samples increased from 1.73 – 2.1 mg/L (22.7%) after a brief boiling period. Fluoride content in raw foods was lowest in cassava roots (4.24 × 10^–2^ mg/g) and highest (1.05 mg/g) in small fishes (Table 4). Fluoride content was higher in porridge (0.47–0.50 mg/g) than any other cooked food samples collected from the two households.

**Table 3:** Fluoride content of raw water samples collected from Gihaya Island, Rwanda (2018, 2019)

	**Fluoride content (mg/L)**				
**Sample**	**1**^**st**^** test**	**2**^**nd**^** test**	**3**^**rd**^** test**	**Mean**	**SD**
*December 2018*					
LK	1.79	1.73	1.73	1.75	0.3
SP	0.14	0.13	0.12	0.13	0.1
HPRW	2.36 × 10^–3^	1.96 × 10^–3^	1.33 × 10^–3^	1.88 × 10^–3^	5.19 × 10^–4^
*July 2019*					
LK 1	1.63	1.64	1.64	1.64	0.006
LK 2	1.63	1.65	1.64	1.64	0.01
LK 3	1.65	1.67	1.67	1.71	0.01
LK 4	1.64	1.64	1.65	1.64	0.006
LK 5	1.64	1.64	1.64	1.64	0
LK 6	1.74	1.70	1.70	1.71	0.02
SP	0.13	0.14	0.12	0.13	0.004
HPRW	0.03	0.02	0.02	0.02	0.006

*LK* Lake Kivu, *HPRW* Health Post Rainwater, *SP* Solar Pump, *SD* Standard Deviation

**Table 4:** Fluoride level in local foods Gihaya Island, Rwanda (2018, 2019)

	**Fluoride content (mg/g)**							
**Samples**	**1**^**st**^** test**	**2**^**nd**^** test**		**3**^**rd**^** test**	**Mean F**^**−**^			**SD**
*Raw foods*								
Beans (fresh)	0.56	0.52		0.64	0.57			0.06
Bean leaves	0.91	0.96		0.98	0.94			0.03
Cassava roots	3.85 × 10^–2^	4.57 × 10^–2^		4.32 × 10^–2^	4.24 × 10^–2^			4.5 × 10^–3^
Cassava leaves	0.84	0.88		0.77	0.82			0.05
Small fishes	1.03	1.08		1.06	1.05			0.02
*Cooked foods*								
Beans HH1^a^	0.19		0.18	0.19		0.19	0.005	
Beans HH2	0.23		0.24	0.24		0.24	0.005	
Porridge HH1	0.51		0.50	0.50		0.50	0.005	
Porridge HH2	0.47		0.47	0.46		0.47	0.005	
Ugali HH1	0.12		0.12	0.13		0.12	0.005	
Ugali HH2	0.20		0.20	0.20		0.20	0	
Green banana HH1	0.20		0.21	0.21		0.20	0.005	
Green banana HH2	0.15		0.15	0.15		0.15	0	
Fish HH1	0.38		0.37	0.36		0.37	0.01	
Fish HH2	0.16		0.16	0.15		0.16	0.005	
Green marog HH1	0.30		0.31	0.31		0.31	0.005	
Other samples								
Grass	0.17		0.16	0.18		0.17	0.01	
Soil	0.25		0.39	0.32		0.32	0.07	

^a^*HH* Household number

## Discussion

Our One Health study confirms the high prevalence of dental fluorosis in children and goats on a remote island in Rwanda where people and animals rely on Lake Kivu water for drinking and cooking. Fluoride levels that exceeded the WHO safe threshold further indicate Lake Kivu as a primary source of dietary exposure; however, most fluorosis cases likely occur from cumulative exposure through water and food. Those severely affected may experience long term consequences such as tooth fracture, pain, and the ability to masticate. Brown staining and pitting common to fluorosis patients contributes to poor self-esteem and stigma [28]. Although not assessed, risk for skeletal fluorosis should be considered when dental fluorosis is observed. Gihaya Island residents are already marginalized by their remote location, poverty, and lack of government services. They require immediate upgrades to their water infrastructure and oral health services to treat those already affected and to protect the next generation.

The prevalence of dental fluorosis among Gihaya Island children (90.7%) was higher than the national average (6%) [21] but was similar to locations in other East African countries in the Great Rift Valley region, which contains active volcanoes that discharge fluoride gas into natural water bodies [29–31]. Lake Kivu is part of this region and is near several volcanoes, likely explaining the high water fluoride content. In neighboring Tanzania, high fluorosis levels have been linked to unsafe water and food tenderizers (magadi) [32]. Scant data on livestock fluorosis is available in Africa. Certain districts in India have reported prevalence levels among cattle (*N* = 563) and goats (*N* = 563) to be 59 and 12%, respectively, where mean fluoride concentrations in water exceeded 1.5 mg/L [33]. These levels differ from our results which demonstrated fluorosis to be more prevalent among goats than cattle but could be explained by differences in signalment, husbandry, water intake and age [34, 35].

Gihaya Island children and livestock owners both reported Lake Kivu as a primary water source for cooking and livestock. However, fluoride levels in Lake Kivu freshwater were far higher than groundwater and rainwater, especially when boiled, and exceeded the WHO recommended threshold level. Subsequent microbial analysis indicated intermediate to very high risk of Lake Kivu samples due to high coliform contamination. Therefore, Gihaya Island residents who boil their water as a measure to avoid diseases caused by waterborne pathogens also increase their exposure to waterborne fluoride. Overall, rainwater and groundwater appeared to be safer choices for drinking and cooking water, but access became limited during the study period. Access to the sole rainwater catchment tank is restricted to health post use and the solar-powered borehole pump changed from a free to a paid service that is not affordable or geographically convenient for all residents. A campaign to sensitize residents fluorosis etiology should emphasize the importance of safe water for overall household health. Obtaining support to improve access to safe water is essential for the health of the community. Short-term, this could include rehabilitation of the two non-functioning boreholes, reducing the price of the solar pump borehole water or investing in de-fluoridation systems. Long-term interventions could include introducing piped chlorinated water from the mainland or installing rainwater catchment systems.

Our analysis of foods commonly consumed on Gihaya Island suggested that raw fishes and cooked porridge contained the highest levels of fluoride. This is not surprising given that fish bioaccumulate chemicals from their surrounding water and porridge is often prepared by boiling cereals in Lake Kivu water. Some commercially processed foods, such as infant formula, can contain high levels of fluoride. The concentration of fluoride would increase further when prepared with water containing high fluoride levels [36, 37]. The relationship between fluoride levels in raw or cooked foods and dental fluorosis is not well documented. The presence of fluoride in all food, soil and grass samples supports our conclusion that people and animals are primarily exposed through Lake Kivu water but also through various foods. More information on dietary habits, food preferences, food frequency, and preparation methods is needed to further estimate the daily fluoride intake due to food versus water.

Dental fluorosis is known to adversely impact the quality of life of those affected [38]. Nearly all children on Gihaya Island were visibly affected by fluorosis and three-quarters were dissatisfied with the appearance of their teeth, suggesting that more information is needed on the social impact of this condition. Restoration of teeth damaged by excess fluoride intake requires access to specific dental services (tooth bleaching, micro-abrasion, veneering and crowning) [39] that are not available on the island. Although such services are available on the mainland, they are cost prohibitive, especially for those without health insurance. Local misperceptions regarding fluorosis etiology, such as the possibility of contagion, affect quality of life and should be mitigated with local education campaigns in high risk and surrounding areas.

This pilot One Health study evaluated fluorosis among all children and livestock meeting the inclusion criteria, and comprehensively evaluated fluoride sources in common food and water sources. The short study duration and use of single examiners for human and livestock oral exams provided consistency in the fluorosis assessments. However, self-reported survey data may have been affected by recall bias or a desire for social acceptability. We developed a modified Dean’s Fluorosis Index to facilitate the assessment of the severity of dental fluorosis in cattle and goats. While helpful in standardizing animal fluorosis scores, it was not pretested and is a limitation of the study design. Additional information on adult fluorosis prevalence, household diet, and socioeconomic factors would provide a more comprehensive picture of this issue and permit calculation of fluoride exposure through various food and water sources. Even with these limitations, the large sample size in relation to the population provides invaluable foundational information regarding the human, animal, and environmental aspects of fluorosis on Gihaya Island.

### Conclusions

This study documented high levels of dental fluorosis in people and goats on a remote island in Rwanda and identified possible sources of environmental fluoride exposure. The physical and emotional consequences of dental fluorosis can be serious, especially in a marginalized community already affected by food scarcity, poverty, and high levels of infectious disease. A multidisciplinary and holistic approach, including oral health practitioners, veterinarians, physicians, water specialists and social scientists is needed to develop affordable, sustainable, and evidenced-based solutions for reducing the burden of fluorosis on Gihaya Island. This includes identifying safe and accessible drinking water sources, treatment for those already affected, and improving public awareness of prevention practices.

## Supplementary Information

**Additional file 1.** Dental Fluorosis Participant Survey

**Additional file 2.** Dental Fluorosis Livestock Owner Survey

## Data Availability

Data is available upon reasonable request to the corresponding author.

## Abbreviations

NA: Not Applicable
SD: Standard Deviation
SPSS: Statistical Package for the Social Sciences
WHO: World Health Organization
