# Supplementary material for: Dental fluorosis among people and livestock living on Gihaya Island in Lake Kivu, Rwanda
Source: One Health Outlook. 2021 Dec 20;3:23. doi: 10.1186/s42522-021-00054-7 (PMC8686390; doi:10.1186/s42522-021-00054-7)
Supplement: Supplementary file 1 — Additional file 1. Dental Fluorosis Participant Survey [file 42522_2021_54_MOESM1_ESM.docx]

**Additional File 1 - Dental Fluorosis Participant Survey**

Date of data collection: ____ / ____ /____(year/month/day)

Household number: ________________

Respondent's initials: _______________

1. Respondent age: ________________

2. Respondent gender

🞎 Male 🞎 Female 🞎 Other

3. How often do you clean your teeth?

🞎 Never, Skip to question 3

🞎 _______/day; 🞎 _______/week 🞎 _______/month

4. Do you use a toothbrush to clean your teeth?

🞎 Yes 🞎 No

Do you use anything else to clean your teeth? If yes, specify _____________________

5. Do you use toothpaste to clean your teeth?

🞎 Yes 🞎 No

If yes, does your toothpaste contain fluoride? 🞎 Yes 🞎 No 🞎 I do not know

6. Where do you most often collect your drinking water? (Check all that apply)

🞎 Lake Kivu 🞎 Water pump 🞎 Other, specify ________________

7. Where do you most often collect your cooking water? (Check all that apply)

🞎 Lake Kivu 🞎 Water pump 🞎 Other, specify ________________

8. How often do you eat the following food (grown and collected on this Island)?

|  | Never | 1/day | 2/day | 3/day | >3/day |
| --- | --- | --- | --- | --- | --- |
| A. Fish/small fishes | 🞎 | 🞎 | 🞎 | 🞎 | 🞎 |
| B. Beans | 🞎 | 🞎 | 🞎 | 🞎 | 🞎 |
| C. Cassava (flour or root) | 🞎 | 🞎 | 🞎 | 🞎 | 🞎 |
| D. Cassava leaves | 🞎 | 🞎 | 🞎 | 🞎 | 🞎 |
| E. Bean leaves | 🞎 | 🞎 | 🞎 | 🞎 | 🞎 |
| F. Other foods _____________ | 🞎 | 🞎 | 🞎 | 🞎 | 🞎 |

9. In the past 12 months, was there a time you needed dental care and did not access it?

🞎 Yes 🞎 No 🞎 Not applicable

If yes, why not?

🞎 Did not have time 🞎 Would cost too much 🞎 No one else was available to care for family

🞎 Other, specify __________________

10. Are you satisfied with the appearance of your teeth?

🞎 Yes 🞎 No

***Next section to be completed by dental technician***

11. Fluoride Index

A. Deans Dental fluorosis examination results (For all teeth)

🞎 Not examined 🞎 No fluorosis 🞎 Questionable 🞎 Very mild 🞎 Mild 🞎 Moderate 🞎 Severe

B. Anterior Fluoride Index examination results (anterior teeth)

🞎 Not examined 🞎 No fluorosis 🞎 Questionable 🞎 Very mild 🞎 Mild 🞎 Moderate 🞎 Severe

12. Teeth decay examination (DMFT/dmft)

| Teeth | Present | Sound | Decayed | Missing | Filled |
| --- | --- | --- | --- | --- | --- |
| 11 | 🞎 | 🞎 | 🞎 | 🞎 | 🞎 |
| 12 | 🞎 | 🞎 | 🞎 | 🞎 | 🞎 |
| 13 | 🞎 | 🞎 | 🞎 | 🞎 | 🞎 |
| 14 | 🞎 | 🞎 | 🞎 | 🞎 | 🞎 |
| … |  |  |  |  |  |
| 85 |  |  |  |  |  |

13. Dental/medical treatment needed:

🞎 Preventive care/no apparent problem

🞎 Early dental/medical care needed (including scaling) – within several weeks

🞎 In need of immediate referral for pain or infection that requires immediate relief – as soon as possible

🞎 In need of immediate referral for other conditions (life threatening condition or other severe condition with oral manifestations - conditions should be specified e.g., NOMA) ______________________________

14. Referral to Community Health Worker or Health Center for medical evaluation

🞎 No 🞎 Yes, specify reason_________________________________
