# Supplementary material for: Dental fluorosis among people and livestock living on Gihaya Island in Lake Kivu, Rwanda
Source: One Health Outlook. 2021 Dec 20;3:23. doi: 10.1186/s42522-021-00054-7 (PMC8686390; doi:10.1186/s42522-021-00054-7)
Supplement: Supplementary file 2 — Additional file 2. Dental Fluorosis Livestock Owner Survey [file 42522_2021_54_MOESM2_ESM.docx]

**Additional File 2 - Dental Fluorosis Livestock Owner Survey**

Date of data collection: ____ / ____ /____(year/month/day)

Household number: ________________

Respondent's initials: _______________

1. Respondent age: ________________

2. Respondent gender

🞎 Male 🞎 Female 🞎 Other

3. Who is responsible for taking care of livestock?

🞎 Respondent 🞎 Other, Specify__

4. Types of animals in household

🞎 Cattle, # _________ ; 🞎 Goats, # _________ ; 🞎 Swine, #_________

| 5. Where do you get your animal's drinking water?  🞎 Lake Kivu 🞎 Water pump 🞎 Other, specify __________  6. Do your animals have access to Lake Kivu during the day?  🞎 Yes 🞎 No  7. Where do your animals graze during the day? _________________  8. Do you give leftover household food to your animals?  🞎 Yes 🞎 No 🞎 I do not know  If yes, what kind of food? ______________  ***Next section to be filled by veterinarian*** | 2/3 |
| --- | --- |

9. Does any animal at this location have fluorosis?

🞎 Yes 🞎 No

10. Owner feedback

🞎 Animal home care advice 🞎 Refer to private veterinarian (reason: _________________)
